# Developing Employment Environments Where Individuals with ASD Thrive: Using Machine Learning to Explore Employer Policies and Practices

**DOI:** 10.3390/brainsci10090632

**Published:** 2020-09-11

**Authors:** Amy Jane Griffiths, Amy Hurley Hanson, Cristina M. Giannantonio, Sneha Kohli Mathur, Kayleigh Hyde, Erik Linstead

**Affiliations:** 1 Attallah College of Educational Studies, Chapman University, One University Drive, Orange, CA 92866, USA; mathu109@mail.chapman.edu; 2 Argyros School of Business and Economics, Chapman University, One University Drive, Orange, CA 92866, USA; ahurley@chapman.edu (A.H.H.); giannant@chapman.edu (C.M.G.); 3 Schmid College of Science and Technology, Chapman University, One University Drive, Orange, CA 92866, USA; khyde@chapman.edu; 4 Fowler School of Engineering, Chapman University, One University Drive, Orange, CA 92866, USA; linstead@chapman.edu

**Keywords:** autism spectrum disorder, machine learning, employment

## Abstract

An online survey instrument was developed to assess employers’ perspectives on hiring job candidates with Autism Spectrum Disorder (ASD). The investigators used K-means clustering to categorize companies in clusters based on their hiring practices related to individuals with ASD. This methodology allowed the investigators to assess and compare the various factors of businesses that successfully hire employees with ASD versus those that do not. The cluster analysis indicated that company structures, policies and practices, and perceptions, as well as the needs of employers and employees, were important in determining who would successfully hire individuals with ASD. Key areas that require focused policies and practices include recruitment and hiring, training, accessibility and accommodations, and retention and advancement.

## 1. Introduction

Research suggests that competitive employment may be difficult to attain for individuals with Autism Spectrum Disorder (ASD). Although companies are beginning to recognize the value of hiring employees with ASD, the academic literature on the benefits and current practices of recruiting people with ASD is limited [1,2]. Providing supportive employment services for adults with ASD is seen as a positive investment. Individuals with ASD can typically gain employment with the right support [3]. Research indicates that employees with ASD have many skills that can contribute a great deal to the workforce. Despite the skill sets of individuals with ASD, the unemployment and underemployment rates for these individuals, as compared to the general population, remains staggeringly low [4,5,6,7,8,9,10]. This discrepancy suggests that it is critical to understand employers’ perspectives and experiences, so that hiring practices and outcomes may be improved for both organizations and employees with ASD.

## 2. Background

Organizations’ interest in hiring neurodiverse individuals, including those with ASD, has increased [2]. This interest is due in part to companies recognizing the value of hiring employees with ASD. Research indicates that employees with ASD typically pay close attention to detail, enjoy certain job tasks that other employees may find repetitive or socially isolating, and bring a different perspective to issues, allowing for innovative solutions to common problems [3,11]. Research also suggests that employees with ASD have high levels of trustworthiness, integrity, and honesty. They are reliable, precise, efficient, and consistent [4,7,12,13].

Additionally, employees with ASD may demonstrate “above standard” workplace performance compared to their counterparts related to increased attention to detail, work ethic, and quality of work [9]. Employees with ASD have been found to have fewer absences and are more likely to arrive at work on time than other employees [12,14,15]). Research has also found that employees with autism have dramatically lower turnover rates than neurotypical employees. Turnover is a large expense for organizations. In some industries, such as software, the turnover rate is close to fifteen percent nationally. Employees with autism have a seven percent turnover rate. The costs of replacing a worker earning less than $50,000 are estimated to be twenty percent of their annual salary [16]. As salaries rise, so do the costs of replacing those employees.

Moreover, an increase in the employment of people with ASD can lead to significant economic benefits to society [3,17,18,19]. A study in Australia found that reducing the unemployment of people with ASD by one-third would lead to a $43 billion increase in the Australian Gross Domestic Product [20]. The Dandelion Employment Program in Australia calculates that every 100 individuals with ASD who were previously unemployed, and who participate in the program for three years, save the Australian government over six million dollars in the form of tax gains, savings in welfare benefits, and savings in unemployment services costs.

Despite these individual positive qualities, and benefits to organizations and society, many individuals with ASD remain unemployed, underemployed, and underpaid. Recent unemployment statistics for adults with ASD reveal that 85% are unemployed (National Autistic Society, 2016). Research has shown that many individuals with ASD have never been members of the labor force [21]. The authors of [10] found that thirty-five percent of young adults with autism have never held a job, been members of the labor force, or attended educational programs after high school [10,21]. A study of 200 transition-age young adults with ASD found that 81% were unemployed [22].

Understanding the factors that contribute to unemployment is critical. The number of people affected by Autism Spectrum Disorder (ASD) is estimated in the tens of millions worldwide and 3.5 million in the United States [2]. Moreover, it is predicted that over the next decade, close to half a million children with ASD will reach adulthood (https://www.cdc.gov/ncbddd/autism/index.html). If they cannot be employed and live independently, the services they will require will place a financial toll on families and society [3]. Supporting an individual with ASD may exceed two million dollars throughout their lifetime [17]. The total cost of ASD support services in the U.S. exceeds 236 billion dollars annually [17]. This number is expected to rise to one trillion dollars by 2025 [4]. There are additional financial and non-financial costs that are difficult to measure, such as income losses for individuals with ASD and their families, as well as the emotional and psychological costs associated with long term unemployment [1].

Research must examine why organizations hire individuals with ASD and the barriers to their employment [2]. The literature on the employment of people with disabilities has found that, although many employers say they are willing to hire those with disabilities, their actual hiring practices do not show efforts in this area [23,24,25]. Employer’s attitudes and perceptions towards people with disabilities, and organizational practices and policies, are two significant barriers to employment success [23]. This paper examines the role of employers’ attitudes, perceptions, organizational practices, and policies on the hiring and retention of employees with ASD.

### 2.1. Research on Employer Attitudes and Perceptions

The literature on employer attitudes and perceptions towards employees with ASD is limited. However, there are studies in the disabilities literature that may lend insight into employers’ attitudes and perceptions of individuals with ASD. Investigators at Cornell University surveyed over 800 private sector employers and over 400 federal sector employers in regard to actual hiring and retention processes for employees with disabilities [26,27].

When considering hiring individuals with disabilities, employers have several concerns. These concerns include potential legal risks, the time and effort to supervise and train, safety issues, the financial burden of accommodations, and the belief that they would never be able to terminate the disabled employee once hired [24,28,29,30,31]. Specifically looking at ASD, the authors of [32] found that employers who do not hire employees with ASD tend to have the following concerns; focusing on the employee’s ability to adapt to work situations, a concern for adverse outcomes, and a lower interest in receiving new information and training. A study focusing on employees with Asperger’s Syndrome (now categorized as ASD), found that employers’ resistance and negative attitudes about the (perceived) need to provide accommodations, concerns about high costs, low productivity, and high turnover, as well as the need to provide outside supports, were associated with lower rates of hiring and higher rates of termination of individuals with ASD [7].

However, research has contradicted employer attitudes and perceptions that hiring adults with ASD may result in a loss of productivity and increased costs associated with workplace modifications and additional training and supervision. A few studies have compared the job performance ratings of employees with and without ASD [3]. Managers tended to rate the job performance of the employee with ASD as average or above average [11,14,33]. The authors of [34] compared employees with and without ASD on the extent to which they met standard requirements for good workplace performance. They found that employees with ASD performed at an “above standard level” regarding attention to detail, work ethic, and quality of work. This study found that employers do not incur additional costs when employing an adult with ASD over and above that of any new employee. They also found that while they may require some workplace modifications, supervision, and training, there is no significant difference between them and their colleagues concerning weekly employment, supervision, and training costs. Twaronite found that those with ASD were able to identify process improvements that cut training time in half (https://wwTw.ey.com/en_us/diversity-inclusiveness/six-ways-to-advance-disability-inclusion-in-your-organization). They also found that quality of work, efficiency, and productivity was equal to their other employees. JP Morgan & Chase Company has employed over 70 individuals with ASD over the past three years (https://www.forbes.com/sites/jpmorganchase/2017/06/05/how-jpmorgan-chases-autism-at-work-program-is-helping-to-win-top-tech-talent/#660a965830bb). Representatives of JP Morgan & Chase Company report that their employees with ASD are producing forty-eight to one-hundred-and-forty percent more work than their neurotypical colleagues (https://fortune.com/2018/06/24/where-autistic-workers-thrive/). They anticipate hiring hundreds more individuals with ASD globally in the coming years. A survey of employers who had hired individuals with ASD found that fifty-seven percent of employers reported no additional costs from hiring an individual with ASD and did not require assistance from tax incentives to hire them (https://askjan.org/topics/costs.cfm). Consequently, at the organizational level, these results contradict employers’ attitudes and perceptions that hiring adults with ASD may result in a loss of productivity and increased costs associated with workplace modifications and additional training and supervision.

### 2.2. Research on Employer Practices and Policies

Research on employers’ practices and policies to support employees with ASD is in its infancy. Although many organizations have created hiring initiatives to hire individuals with disabilities, some are beginning to focus on explicitly hiring individuals with ASD [27]. Organizational practices and policies may influence all stages of employment. A holistic approach to hiring for employees with ASD should be encouraged [35]. To better assist organizations in improving their employment practices, the barriers and facilitators to hiring individuals with ASD must be understood [2].

Much attention has focused on how the traditional selection interview may operate as a barrier to employment for individuals with ASD. The authors of [36] note that one of the obstacles to hiring neurodiverse employees is the traditional interview process. Current recruitment interview processes have not been found to provide adequate accommodations for neurodiverse individuals [37,38]. The authors of [36,39] found that the employment interview poses a unique barrier for individuals with ASD. Their research found that it is difficult for individuals with autism to navigate the social cues present during an interview. Research has found that individuals with ASD experience high anxiety during employment interviews [4,40]. A study of adults with ASD participating in an interview process found that 100% of the participants found that their high levels of anxiety negatively influenced their experience with the interview process. The participants also reported that their anxiety negatively affected their communication and performance in the interview (https://search.proquest.com/openview/b2bf39c3621955296526edd36f51dde6/1?pq-origsite=gscholar&cbl=18750&diss=y). The study also found that all participants viewed the interview as a negative experience due to issues of verbal and nonverbal communication, the process of the interview, and anxiety. For example, when companies use online applications, candidates may not be able to complete the application without adaptive technology, such as talk-to-text software. This can prevent a potential employee from even being eligible for an interview. Some organizations replace traditional interviews with opportunities for applicants with ASD to engage in a job trial or to demonstrate their skills with alternative methods [27].

The work in [35] found that, in Australia and Sweden, employers reported that knowledge and understanding of ASD, work environment, and job match led to successful employment of individuals with ASD. The authors of [35] noted that, when employees had a working knowledge of the needs of individuals with ASD, they fostered successful workplace relationships, minimized misunderstandings, and increased communication. The authors of [41], who interviewed employment support service providers, found that accommodations need to begin during the hiring process, not after the person has been hired. Some organizations may benefit from using support specialists in the hiring process. Support specialists help employers to see how the organization can successfully hire individuals with disabilities, including ASD. They will also assist them with training other employees and needed accommodations [42,43,44,45,46,47,48]. When education comes directly from employment support specialists during the hiring process, employers feel more confident about hiring employees with ASD. Employers reported that having an employment support specialist allows them to have someone to rely on. Employers can then ask disability-related questions, understand accommodation needs, and ease legal concerns [31,42,48,49,50]. Research is needed that examines organizational policies and practices that lead to successful employment outcomes for individuals with ASD [2].

### 2.3. Current Study

The literature explores employer perspectives and practices in regard to hiring employees with disabilities. There is limited research on employer factors that contribute to successful hiring and retention practices of employees with ASD, specifically. In this study, we used K-means clustering, a form of unsupervised machine learning, to categorize companies in different clusters based on their response to an online survey. This analysis allowed us to assess and compare aspects of businesses that successfully hire employees with ASD versus those that do not. Clustering algorithms aim to partition the dataset into groups (clusters) in which members of each group are similar to those in their cluster and dissimilar to those in other clusters. This type of analysis allows for a better understanding of the characteristics of those companies that are more successful in hiring employees with ASD.

## 3. Data

An online survey instrument was developed to assess employers’ perspectives on hiring candidates with “High Functioning” Autism Spectrum Disorder (HFASD). This term was used to provide a brief descriptor of a particular group of individuals with ASD (i.e., those with average to above-average cognitive skills). However, the term HFASD can be problematic, as the term suggests those with average or above cognitive skills perform well in other functional areas, while the evidence indicates this is a poor predictor of functional skills [51]. To ameliorate some of these concerns, while still maintaining a “short descriptor”, investigators clearly defined what was meant by HFASD as it related to the survey. Example behaviors based on the Diagnostic and Statistical Manual of mental disorders, 5th edition [52] were described in the survey and included areas of difficulty in functional domains. Specifically, for the purposes of this study, a person with HFASD is defined as someone who has identified themselves as having Autism, Aspergers, or HFASD has approximately average intellectual ability (when compared to peers) but may have marked difficulties in social interactions, including communication. The employee may have difficulty initiating or responding to social interactions, or may not seem interested in interacting with colleagues or customers. The person may be able to have a conversation when necessary, but may have difficulty keeping the conversation going or knowing how and when to end the conversation. The person may require a structured environment and/or schedule and may not deal well with change. The person may have difficulty with organization and planning.

A review of the literature was conducted to identify related studies across various stakeholders [53,54] to develop a basic framework and a list of questions. Questions used to assess these perspectives were organized into several categories including employer background and characteristics of employees; policies and practices related to recruitment and hiring; training, accessibility, and accommodations; and retention and advancement; barriers and facilitators to hiring individuals with ASD; and finally, the employer needs to improve hiring outcomes for individuals with ASD.

Employers from five local businesses agreed to participate as early reviewers and provided feedback about content as well as readability. A staff member from one local business volunteered to take the survey, along with the authors, and read aloud and answered each question on the computer and verbally. The volunteer asked questions for clarification and provided feedback during the process. In addition, the research team completed the survey multiple times, taking on varying respondent perspectives (e.g., employers with experience in hiring individuals with ASD, employers who had no experience) to assess whether the survey branching logic was appropriate; also discussed were potential answers, the survey flow, and whether the questions were sequenced in a logical order based on earlier responses. All pretest feedback was considered and resulted in multiple revisions. After obtaining Institutional Review Board approval, the survey instrument was finalized and placed in an electronic survey platform (Qualtrics), and a unique resource locator (URL) was created.

To access a broad range of employers across a larger geographical region, the team utilized the Qualtrics research services to recruit survey respondents. An invitation was sent out through their platform. The request to complete the survey included a statement regarding the researchers’ interest in the employer’s opinions on organizational practices, policies, and needs, related to employing people with ASD. However, it was made clear that the employer did not have to have experience hiring an individual with ASD to participate. Respondents were selected if they had a significant role in hiring decisions for their company. The survey link was active and open for three weeks. Participants were able to direct any questions or concerns to the authors; however, no questions or concerns were received.

The survey research methodology involved a traditional analytical process. The researchers had knowledge only about the demographic categories that the respondents provided, and no other identifying information was provided. Quantitative and qualitative data were collected via the survey. Quantitative data were obtained primarily through forced-choice or ranking questions. For most items, standard survey nomenclature (e.g., Likert scales) was used. Specifically, for the policy- and practices-related questions, respondents were asked if a particular policy or practice was not in place, being considered, in place but not effective, in place and somewhat effective, or in place and very effective. When asked about barriers and facilitators, employers were given a range of options to choose from, and an “other” or short text box was available for many questions so that respondents could provide a more detailed response [55]. For example, when asked, “Do any of the following pose a barrier to employment or advancement for people with ASD in your organization?” Respondents could select all that apply from a range of options including (but not limited to) cost of accommodations, cost of training/additional supervision, attitudes/stereotypes, lack of requisite skills among individuals with ASD, productive and performance of an individual, etc. All of the response options are based on previous research described above. When explicitly asked about employer needs related to hiring individuals with ASD, employers were asked a variety of questions about training, tax incentives and support, and experience with incentive and support programs. For example, employers were asked, “If an agency were to provide training and support for employing people with ASD, what would make it worthwhile for your organization to utilize the training?” Employers were able to select all applicable response options including the training would be free of cost, the training would be in partnership with a well-respected community organization concerning HFASD, and the training would be tailored to my company’s needs. They were also asked if they would be willing to pay for training to assist their company in hiring individuals with ASD. The results of the quantitative data analysis are reported in this paper.

The instrument consisted of 50 to 80 questions, depending on the participant’s hiring experience. Specifically, the number of items offered to each respondent varied based on his or her experience with hiring individuals with High Functioning ASD (HFASD). It took approximately 20 min to complete the survey online, and a total of 285 respondents completed the online survey. Of the 285 respondents, 166 (58%) indicated they had hired at least one individual with HFASD in the past five years. Of the 285 respondents, 120 possessed a high school diploma, 36 held an associates degree, 107 held a bachelors degree or higher, and the remainder indicated “other” as their highest level of education. Of the respondents, 14 worked for organizations with 15 or less employees, 7 at organizations with 16–49 employees, 26 at organizations with 50–99 employees, 26 at organizations with 100–499 employees, 13 at organizations with 500–999 employees, and the remainder at organizations with 1000 or more employees.

To create the data matrix, 41 questions and sub-questions from the survey were used to create a binary variable for each item. For each variable, a 1 indicated a favorable response in relation to HFASD. The variables were then broken into five categories: (1) Hiring (*n* = 15), (2) Training (*n* = 8), (3) Accommodations (*n* = 8), (4) Retention (*n* = 10), and (5) Perceptions (*n* = 18). To reduce the dimensions of the dataset, the average score for each respondent in each of the four categories was calculated. Creating an average score for each category also helped to normalize the data. Because the number of variables differed among the categories, using a count (as opposed to an average score) would put more weight on the categories with more variables. For example, if a respondent had a favorable response for seven of the Hiring variables, he or she would have a Hiring score of 7/15 = 0.47. After assessing the cluster model, all clusters had virtually the same Perception score, which led to overlapping clusters. Thus, the Perception scores from the final data matrix were excluded. Having a dataset with large dimensions often leads to a sparse dataspace (all objects may seem dissimilar in this space) and analysis results that may be true with higher dimensionality but that will not necessarily hold in a lower dimensional space. Thus, the final data matrix was a 285 × 4 dimensional matrix that represents the average scores of 285 employers’ responses to the 41 questions mapped to 4 categories. The variable descriptions and survey averages for each category can be found in Table 1, Table 2, Table 3 and Table 4.

## 4. Methods

K-means is an unsupervised learning algorithm that is capable of discerning latent clusters in data [56]. We chose this algorithm for our analysis based on data visualizations which showed no irregular shapes or non-homogeneous behavior which would require a more sophisticated technique. Unlike supervised learning (classification), which requires labeled data to measure the accuracy of prediction, unsupervised learning methods are instead assessed on the “goodness” of the clusters identified using one or more common quantitative metrics, such as silhouette score. K-means requires a single parameter, *k*, as input, which represents the number of clusters to be fit to the data. The algorithm then proceeds as follows.

1. A centroid (mean) for each of the *k* clusters is assigned by randomly selecting a data point from the data.
2. Every other data point is assigned to the cluster whose centroid is closest. Distance can be calculated using any valid distance metric, with Euclidean and Cosine distance being popular choices.
3. The centroids are reevaluated by averaging each data point assigned to a specific cluster.
4. Steps 2 and 3 are repeated, alternating between assigning data points to their nearest centroids, and then reevaluating the value of the centroids based on the new assignments. When the centroids no longer change, or the cluster assignments for the data points become static, the algorithm terminates.

For the work presented here, we found the most likely number of clusters to be five. This was calculated by using the gap statistic [57], a common technique for assessing the number of latent clusters in unsupervised machine learning. This was further validated using the Hopkins statistic, which denotes the overall likelihood that the data can be partitioned into clusters. For the data presented here, the Hopkins score was 0.72, indicating that the dataset exhibits strong clustering tendency.

With clustering, the lack of labeled data means there is not an independent data set to validate accuracy. Instead, silhouette scores [58] were used to measure how well the clusters explained the latent structure of the data. Silhouette scores quantify how similar a data point is to the other points in its cluster compared to those represented in other nearby clusters. The silhouette scores vary from −1 to +1, with values close to 1 suggesting that the point is well clustered and a negative silhouette as suggesting that the data point likely should not belong to its assigned cluster. The mean silhouette score for our model was 0.54.

## 5. Model Results

**Figure 1:**
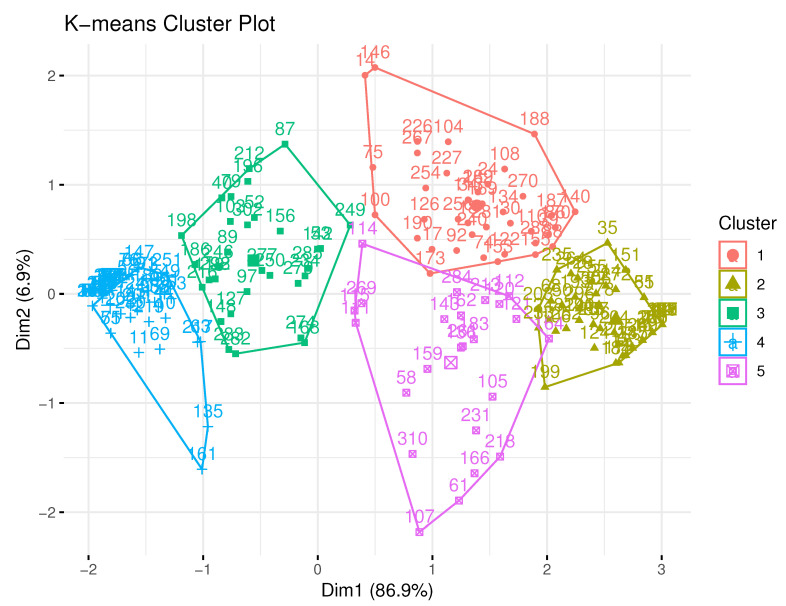
Employer K-means cluster plot.

The data from the complete set of respondents were partitioned into five clusters and visualized in Figure 1, with average category scores listed in Table 5. The remaining figures (presented according to cluster) provide a visualization of the individual cluster average category scores, with the last (presented at the end of this section) that shows all graphs on the same axis for comparison.

### 5.1. Cluster 1

The 41 employer respondents in Cluster 1 (14.0% of sample) had only 29.0% in a management/owner role, while 54.0% were in a human resource/recruiting position. Although employers in Cluster 1 had the lowest five-year hiring rates (24.0% vs. survey average 58.0%) for individuals with high functioning ASD, they are able to offer full-time employment to those they do hire. Almost all, 98.0%, of the employers in Cluster 1 have high functioning ASD employees employed full time. In the past two years, 63.0% of employers in Cluster 1 have hired more than 11 employees.

This cluster had employers in 19 out of the 24 different industries listed and a company size of 218 employees on average (survey average 285 employees). Most respondents in all clusters and the survey were representing health care/social assistance employers. Only the health care/social assistance and construction industries represented at least 10.0% of Cluster 1. A college degree is required by 32.0% of employers in Cluster 1, and 63.0% require a high school diploma for employment. Employers in this cluster had 5.0% in the Southwest, 12.0% in the Northeast, 22.0% in the Southeast, 24.0% in the Midwest, and 34.0% located in the West.

Employers in this cluster do not have policies and practices to foster hiring, retaining, or training employees with high functioning ASD (significantly lower than survey averages for all variables). No employers in Cluster 1 had a hiring initiative for high functioning ASD or a job-related training program for employees with high functioning ASD (compared to 53.0% and 56% of survey respondents, respectively).

**Figure 2:**
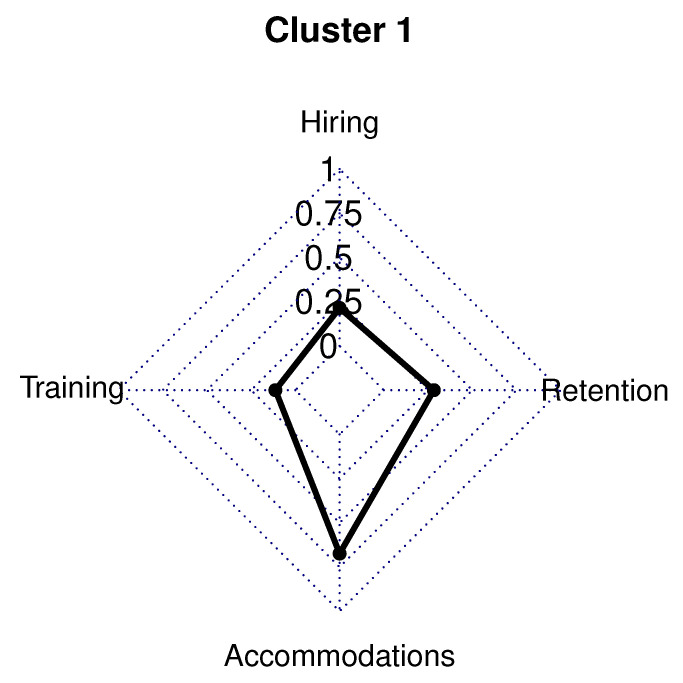
Cluster 1 radar graph.

Employers in this cluster have most accommodations in place for high functioning ASD comparable to the survey averages. Only the following four accommodation variables were significantly higher than the average for the survey. More than 80.0% of employers in Cluster 1 allow an employee to exceed the maximum duration of medical leave as an accommodation, have an established grievance procedure to address reasonable accommodation issues, have a designated office to address accommodation questions, and provide advance notice to job applicants that reasonable accommodations are provided during the job application process (Figure 2).

### 5.2. Cluster 2

The 32 employer respondents in Cluster 2 (11.0% of sample) had 44.0% in a management/owner role, while 53.0% were in a human resource/recruiting position. Respondents in Cluster 2 had a five-year high functioning ASD hiring rate above the survey average (66.0% vs. survey average 58.0%). Cluster 2 had 72.0% of respondents who were involved in the hiring process for less than 10 years (survey average 64.0%). In this cluster, 72.0% of employers have hired over 11 employees in the last 2 years.

This cluster had employers in 18 out of the 24 different industries listed and had a company size of 400 employees on average (40.0% higher than the survey average of 285 employees). Cluster 2 had the highest number, 24.0%, of employers having over 1000 employees compared to the other clusters. Only the insurance/finance (12.0%) and health care/social assistance (16.0%) industries represented at least 10.0% of Cluster 2. A high school diploma is required by 47.0% of employers in Cluster 2, and a college degree is needed for 50.0% of employers in this cluster. Employers in Cluster 2 had 3.0% in the Southwest, 16.0% in the Midwest, 25.0% in the West, 28.0% in the Northeast, and 28.0% in the Southeast.

**Figure 3:**
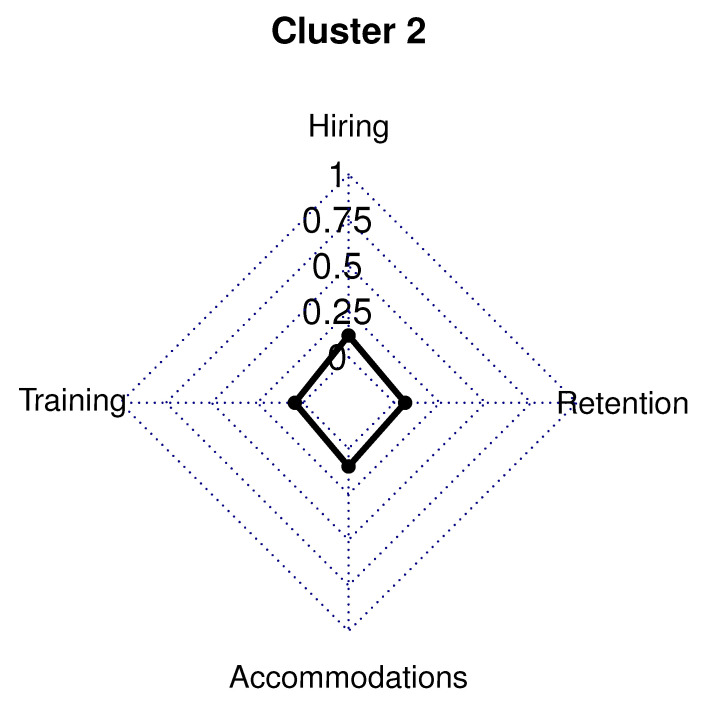
Cluster 2 radar graph.

Cluster 2 employers had most hiring practices similar to the survey averages. Of these employers, 75.0% had senior management that exhibited a strong commitment to high functioning ASD hiring and recruitment (survey average of 56.0%); however, only 25.0% of employers in this cluster require suppliers/subcontractors to follow disability non-discrimination requirements (survey average of 56.0%). Cluster 2 have employers who have human resource staff and supervisors training on high functioning ASD sensitivity and awareness approximately 20.0% more than the survey average. However, only 19.0% do the training internally (survey average of 60.0%). Employers in Cluster 2 also impart training on effective inclusion and interviewing practices for employees with high functioning ASD approximately 20.0% more than the average for survey. In addition, this cluster had about 20.0% higher instances of favorable policies and procedures regarding accommodations and accessibility than the averages for the survey. More than 90.0% of Cluster 2 employers have a defined career path for every employee, have opportunities for advancement for employees with high functioning ASD, and invite employees to confidentially disclose whether they have a disability (Figure 3).

### 5.3. Cluster 3

The 62 employer respondents in Cluster 3 (22.0% of sample) had 45.0% in a management/owner role, while 42.0% were in a human resource/recruiting position. Employers in Cluster 3 had a five-year high functioning ASD hiring rate below the survey average (42.0% vs. survey average 58.0%). Cluster 3 had 52.0% of respondents who were involved in the hiring process for less than 10 years (survey average 64.0%). In this cluster, only 42.0% of employers have hired over 11 employees in the last 2 years.

Cluster 3 had employers in 15 out of the 24 different industries listed and had a company size of 108 employees on average (62.0% lower than the survey average of 285 employees). Only the construction (11.0%), Other (13.0%), and health care/social assistance (19.0%) industries represented at least 10.0% of Cluster 3. A high school diploma is required by 55.0% of employers in Cluster 3, and a college degree is needed for only 29.0% of employers in this cluster. Over half of the employers in Cluster 3 were located in the eastern United States. This cluster had 6.0% in the Southwest, 15.0% in the Midwest, 18.0% in the West, 29.0% in the Northeast, and 35.0% in the Southeast.

Cluster 3 only had two policies and procedures, in any category, that were within 40% of the average for the survey: 47.0% do not automatically exclude applicants with a large gap in employment (76.0% survey average), and 45.0% do not automatically exclude applicants with a history of unemployment (79.0% survey average).

Employers in this cluster had very few favorable hiring policies and procedures in place for individuals with high functioning ASD. Only the following three hiring variables were in place for more than 10.0% of employers in Cluster 3: 11.0% actively recruit individuals with high functioning ASD, 13.0% require suppliers/subcontractors to follow disability non-discrimination requirements, and 46.0% do not automatically exclude applicants with a gap in employment or a history of unemployment. The single training variable present in more than 10.0% of Cluster 3 employers was the 23.0% that train supervisors on the legal requirements related to disability, non-discrimination, and accommodation.

Cluster 3 employers also had few favorable accommodations and accessibility policies and procedures in place for individuals with high functioning ASD. Only the following two accommodations variables were in place for more than 10.0% of employers in Cluster 3: 23.0% allow employees to exceed the maximum medical leave duration, and 19.0% evaluate pre-employment occupational screenings to verify they are unbiased.

**Figure 4:**
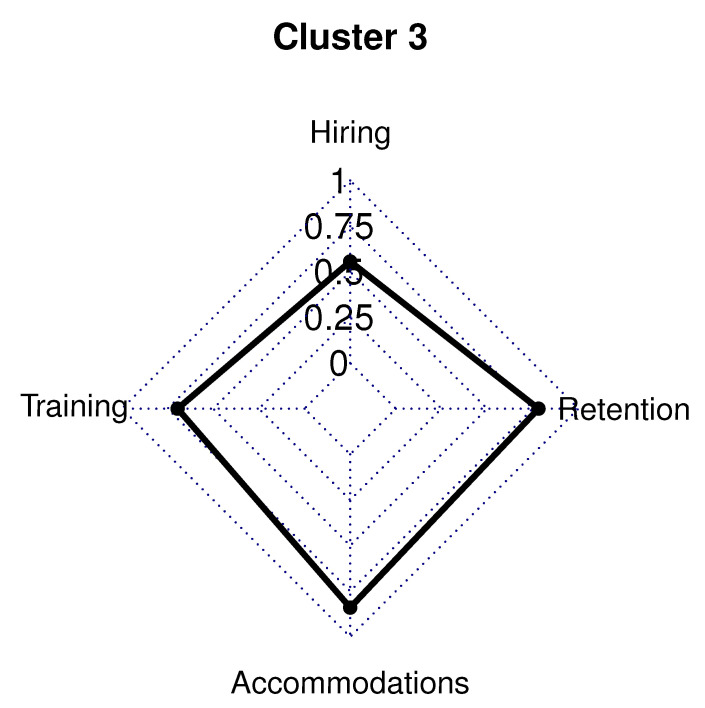
Cluster 3 radar graph.

This cluster only had two favorable policies related to retention of employees with high functioning ASD present in more than 10.0% of employers: 25.0% invite employees to confidentially disclose whether they have a disability, and have a defined career path for all employees (Figure 4).

### 5.4. Cluster 4

The 28 employer respondents in Cluster 4 (8.0% of sample) had 33.0% in a management/owner role, while 67.0% were in a human resource/recruiting position. Employers in Cluster 4 had a five-year high functioning ASD hiring rate below the survey average (46.0% vs. survey average 58.0%). Cluster 4 had 67.0% of respondents who were involved in the hiring process for less than 10 years (survey average 64.0%). In this cluster, 54.0% of employers have hired less than 11 employees in the last 2 years.

Cluster 4 had employers in 13 out of the 24 different industries listed and had a company size of 250 employees on average (survey average of 285 employees). Only the retail trade (12.0%) and health care/social assistance (29.0%) industries represented at least 10.0% of Cluster 4. A high school diploma is required by only 38.0% of employers in Cluster 4, and a college degree is needed for 63.0% of employers in this cluster. Over half of the employers in Cluster 4 were located in the eastern United States. This cluster had 8.0% in the Midwest, 13.0% in the Southwest, 21.0% in the West, 29.0% in the Northeast, and 29.0% in the Southeast.

Most employers in in Cluster 4 had training policies and procedures in place within 10.0% of the survey averages. There were four training policies in place in ~20.0% more employers than the survey averages: work with an agency to provide the support needed for working with individuals with high functioning ASD, train human resource supervisors and staff on effective inclusion and inclusion practices, and offer sensitivity and awareness training internally.

The majority of employers in Cluster 4 did not have policies in place to stimulate accommodating, hiring, or retaining individuals with high functioning ASD. Nearly all hiring procedures related to employees with high functioning ASD were approximately 20.0–40.0% below the average for the survey. Only one hiring variable had an average comparable to to the survey average: 50.0% actively recruit employees with high functioning ASD (53.0% survey average)

**Figure 5:**
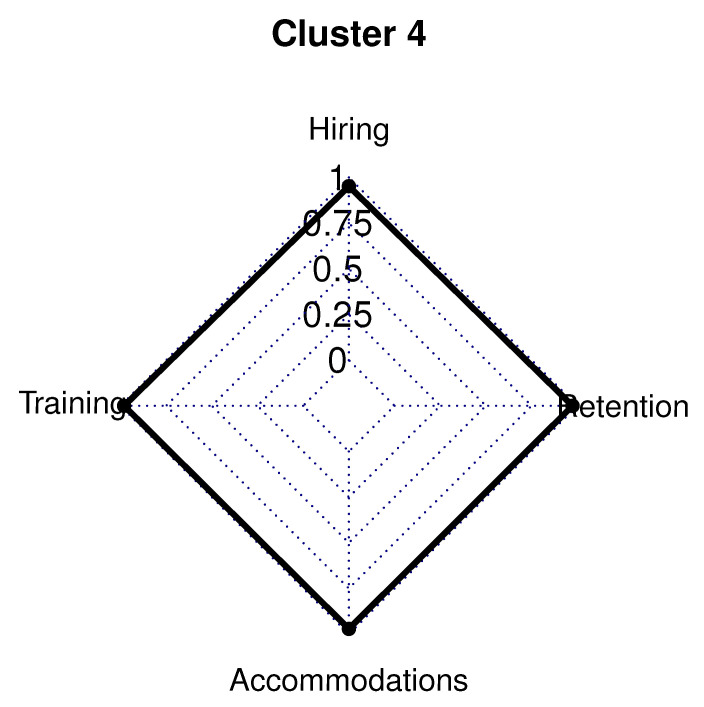
Cluster 4 radar graph.

All accommodations and accessibility variables were approximately 30.0–44.0% below the survey average for employers in Cluster 4. All but one retention policies were 30.0–45.0% below the survey average: 54.0% of employers provide a defined career path for all employees (Figure 5).

### 5.5. Cluster 5

Cluster 5 is the largest cluster of the model. The 126 employer respondents in Cluster 5 (44.0% of sample) had 40.0% in a management/owner role, while 56.0% were in a human resource/recruiting position. Employers in Cluster 5 had a five-year high functioning ASD hiring rate far above the survey average (86.0% vs. survey average 58.0%). Cluster 5 had 75.0% of respondents who were involved in the hiring process for less than 10 years. This is consistent with the work in [59], which found employers newer to the hiring process were more likely to hire an individual with high functioning ASD. In this cluster, 71.0% of employers have hired over 11 employees in the last 2 years.

This cluster had employers in all 24 different industries listed and had a company size of 375 employees on average. The average company size is higher than the survey average of 285 employees, but comparable to the that of Cluster 2 (400 employees). Cluster 5 had 19.0% of employers with over 1000 employees compared to the other clusters. Only the educational services (12.0%) and health care/social assistance (20.0%) industries represented at least 10.0% of Cluster 5. A high school diploma is required by 29.0% of employers in Cluster 5, and a college degree is needed for 64.0% of employers in this cluster. Most employers in this cluster were located in the eastern United States. Employers in Cluster 5 had 7.0% in the Southwest, 15.0% in the West, 18.0% in the Midwest, 22.0% in the Northeast, and 40.0% in the Southeast.

Cluster 5 employers had very high rates of favorable policies and practices in place for individuals with high functioning ASD. The following three polices were in place for *all* employers in this cluster; have a company-wide initiative to higher employees with high functioning ASD, offer a job-related training program for individuals with high functioning ASD, and include individuals with high functioning ASD explicitly in their diversity and inclusion plan.

**Figure 6:**
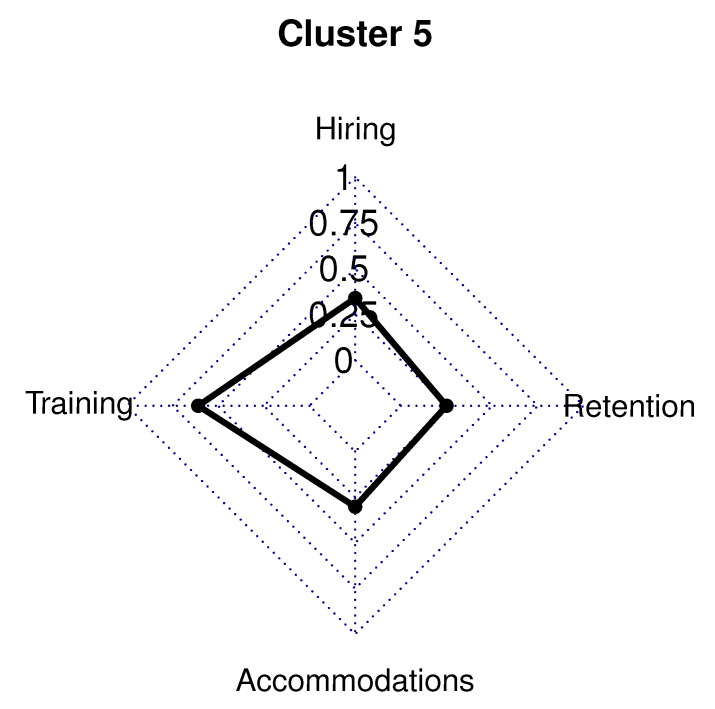
Cluster 5 radar graph.

All but two hiring policies and procedures in place for more than 90% of the Cluster 5 employers: 82.0% have relationships with community organizations that promote the employment of individuals with high functioning ASD, and 88.0% actively recruit individuals with high functioning ASD. Every accommodation, retention, and training policies included in the survey were in place for almost all, 94.0%, of Cluster 5 employers (Figure 6).

### 5.6. Summary of Cluster Comparisons

Most of the respondents were employed in Human Resources and/or Recruiting, which was consistent across all clusters. Cluster 5 had the highest high functioning ASD hiring rate for the past five years, at 86.0%, and, surprisingly also had the most cluster members. The two largest clusters (3 and 5) also had the most extreme average scores for each category. This could indicate that employers tend to have either extremely favorable policies and practices in place or none at all. The most prevalent industry in the survey and all clusters was health care/social assistance, but the cluster with the highest rate of high functioning ASD employment (Cluster 5) did not have the highest rate of health care/social assistance.

**Figure 7:**
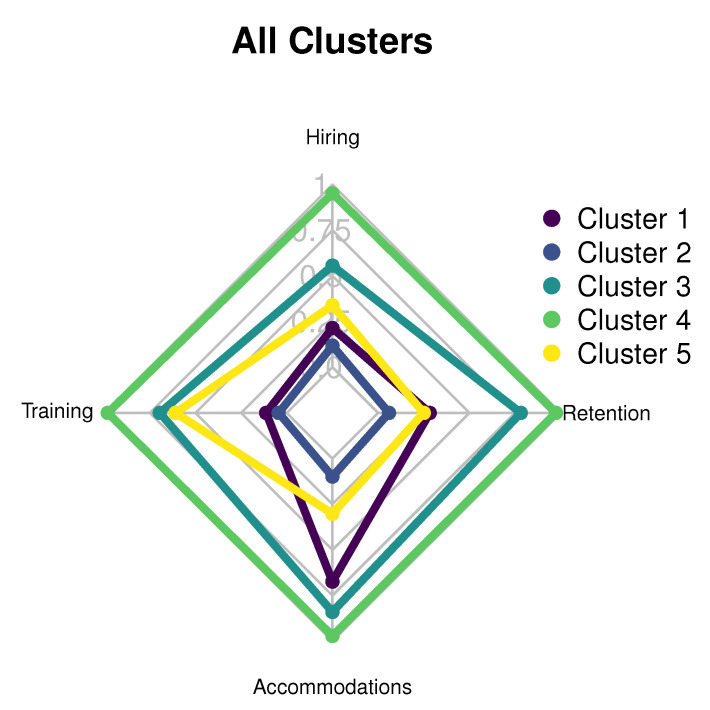
Radar graph for all clusters.

Only two clusters (2 and 5) have employment rates above the survey average of 58.0%. These also are the only two clusters with an average company size above the survey average. Although large companies hire more employees, over 50.0% of employers in both of these clusters require a college degree for entry-level jobs. Clusters 1 and 3 had the lowest rates of hiring high function ASD (24.0% and 26.0%, respectively) and are the only two clusters with rates for requiring a college degree below 50.0% (32.0% and 29.0%, respectively). Figure 7 shows all graphs on the same axis.

## 6. Discussion

Although Cluster 3 had the lowest overall score across four categories (hiring, training, accommodation, and retention), Cluster 1 had the lowest hiring rate for individuals with ASD. For this reason, Cluster 1 was compared to Cluster 5, which had the highest hiring rate for individuals with ASD. Cluster 5 also offered a sharp contrast to Cluster 1 in the number of policies in place in support of employees with ASD. Comparing the company structures, policies and practices, perceptions, and needs of employers and employees within these two clusters allows a determination of best practices for companies that are looking to improve their employment rates of individuals with ASD.

### 6.1. Company Structure, Policies, and Practices

Given that company size and job structures affect hiring practices, company data across the two clusters are included. For size, 58.5% of companies in Cluster 1 had more than 50 employees, compared to 75.3% of Cluster 5 companies. This is important to note, as larger companies may have more opportunities to hire employees with ASD. There also was a difference between job availability and skill sets needed for the different clusters. In Cluster 5, over half of the entry-level jobs require a bachelor’s degree (53.2%), whereas, for Cluster 1, only 24.4% require a bachelor’s degree, and most require a high school diploma (63.4%). This may be affected by the fact that 70.7% of employers in Cluster 5 work directly with universities to hire employees with HFASD.

In comparison to Cluster 1, companies in Cluster 5 provide their employees with ASD more opportunities for professional growth. For example, 73.0% of Cluster 5 employees with HFASD are paid more now than when they started, and 65.0% of Cluster 5 employees with HFASD have been promoted or had an increase in job responsibilities, versus just 7% in Cluster 1. Further, 50.0% of respondents in Cluster 5 reported that their employees disclosed their HFASD during the application or interview process, rather than after being hired (37.5%) or not at all (12.5%) This could reflect a tendency of the companies in Cluster 5 to be responsive and supportive to a disability disclosure during the interview process.

In Cluster 5, 65.1% of companies created incentives to work at their companies by offering credit-based internships for employees with high functioning ASD, compared to 7.3% in Cluster 1. In addition, about half of the organizations in Cluster 5 show purposeful initiatives to hire employees with high functioning ASD. In contrast, every company in Cluster 1 responded that they did *not* have any purposeful initiatives to hire individuals with high functioning ASD. This indicates that there are no current or future plans for initiatives related to hiring people with high functioning ASD, in contrast to Cluster 5, which included positive responses to many of the initiatives to specifically hire, support, and retain employees with high functioning ASD. One of the few initiatives that 58.6% of Cluster 1 organizations did have was advance notice to job applicants that reasonable accommodations are provided during the job application process. Cluster 1 responses, however, indicated that employers did not offer much beyond this, whereas 81.0% of organizations in Cluster 5 had the accommodations in place. Although none of the companies in Cluster 1 reported having hiring initiatives for employees with high functioning ASD, Cluster 5 reported having specific initiatives to hire employees with high functioning ASD to create a more inclusive workplace (21.0%), because they recognize the skills of employees with high functioning ASD (21.0%), to increase the company’s reputation benefits (7.0%), and to decrease employee turnover (2.0%).

Employers in Cluster 1 provide far fewer accommodations than do those in Cluster 5. For example, 81.7% of Cluster 5 employees state that they have a centralized accommodations fund to specifically provide accommodations for employees with disabilities, compared to 39.1% in Cluster 1. Further, just 2.4% of employers in Cluster 5 reported *not* having a designated office or person to address accommodation questions compared to 17.1% in Cluster 1 who do not have a designated accommodation office or person. These data indicate that it may be necessary for employers to provide a menu of accommodation options for their employees, rather than using a one-size-fits-all approach.

Because all respondents in Cluster 1 indicated that they did not have initiatives to specifically hire employees with high functioning ASD, their survey was auto-formatted to skip questions regarding these specific hiring practices. Although this does not allow for comparisons of specific high functioning ASD hiring practices between Clusters 1 and 5, the difference in responses indicates that Cluster 1 companies did not have any initiatives to specifically hire employees with high functioning ASD, while Cluster 5 companies did.

Many employers in Cluster 5 said that they have relationships with community organizations that promote the employment of people with high functioning ASD, with 81.8% that reported that such a program was already in place, and 17.5%, that such a program was being considered. Approximately, 70.7% of Cluster 5 also reported having relationships with universities, with 23.0% that noted that such a program was being considered, and 4.0%, that such a program was not currently in place.

### 6.2. Perceptions and Attitudes

Questions related to employer attitudes demonstrated, in Cluster 5, a company belief in the high competency of their employees with high functioning ASD. For example, 74.6% of employers in Cluster 5 reported being likely to hire employees with high functioning ASD versus 17.1% of Cluster 1. In Cluster 5, 65.1% of employees with high functioning ASD have been promoted or taken on additional job responsibilities, whereas only 7.3% of Cluster 1 had. Generally, employers in Cluster 5 reported having supportive coworkers, reflecting a positive work environment as well as an overall positive experience in working with employees with high functioning ASD, with 20.6% rating their experience as positive and 53.2% as very positive. The majority (78.0%) of respondents in Cluster 1 did not have experience with working with high functioning ASD; thus, comparison data were not available.

The data from Cluster 5 demonstrate an inclusive culture in which high functioning ASD training is offered to employees. Employers offer HR and staff training on inclusive practices (80.2%) and legal requirements (77.8%), high functioning ASD sensitivity and awareness training (72.9%), and training related to effective interviewing of potential employees with high functioning ASD (83.4%). In Cluster 1, 87.8% do not offer high functioning ASD sensitivity and awareness training, 87.8% do not have training in effective interviewing of potential employees with high functioning ASD, 63.4% do not require training related to legal requirements and nondiscrimination accommodation, and 90.2% do not train staff on inclusive workplace practices of people with high functioning ASD.

The biggest fears of hiring someone with high functioning ASD were the same for both clusters: high functioning ASD employees would have behaviors that put themselves or others at risk (Cluster 1: 24.0%; Cluster 5: 29.0%) and employees with high functioning ASD would not perform well (Cluster 1: 24.0%; Cluster 5: 23.0%). Despite having the same fears, Cluster 5 businesses responded positively to hiring employees with high functioning ASD.

### 6.3. Needs of Employers and Employees

When asked about obtaining additional training in hiring practices, respondents in Clusters 1 and 5 stated that they would prefer that training in hiring employees with high functioning ASD be free, but only 43.9% of Cluster 1 were willing to pay, whereas 80.0% of Cluster 5 were willing to pay.

Having a supportive environment helps to alleviate stress and boost productivity [35]. In Cluster 5, the majority of high functioning ASD employees seem to need some form of support, but these companies appear to be prepared to provide that support. Cluster 1 has a far lower perceived need of support for high functioning ASD employees, but perhaps employers are simply unaware of the support needed or less open to offering extra support. For the level of support that their employees with high functioning ASD need, 75.6% of Cluster 1 companies did not respond, indicating a lack of experience with high functioning ASD employees. Of those who did respond, their answers were that their employees with high functioning ASD required: no support (7.3%), some support (12.2%), substantial support (2.4%), or very substantial support (2.4%). In Cluster 5, 12.1% did not respond, but the remaining responded: no support (6.3%), some support (30.2%), substantial support (28.6%), or very substantial support (19.8%). In Cluster 5, 47.6% reported that high functioning ASD employees are in a job-related training program, whereas, in Cluster 1, employers responded that none of their employees with high functioning ASD is in a job-related training program. After considering the notable factors in the in-depth cluster comparisons and reviewing the literature in the field, the researchers identified best practices in employment that would likely lead to the successful hiring of individuals with ASD. These components were organized under a summary of best practices for improving employment, to assist with applying the study outcomes to the field and presented in Table 6.

## 7. Conclusions

This study supports some of the findings of the research on employer perspectives on hiring employees with ASD [7,25,32], but also expands upon Lindsay et al.’s study by using machine learning to analyze trends in businesses that have successfully hired and retained employees with ASD [27]. To foster such diversity and inclusion, it is critical to understand the needs of both the employees and employers. The results of this analysis may be utilized to make suggestions for stakeholders who are working to make improvements to the employment of individuals with high functioning ASD.

The comparison of clusters of employers with the highest and lowest rates of employing individuals with ASD revealed company policies and practices that can be effective for hiring, training, and retaining employees with ASD. The results show that purposeful diversity initiatives and relationships with community organizations that promote ASD are likely effective in increasing employment for those with ASD. Offering disability and diversity training is also helpful for understanding the needs of employees with ASD. These steps may affect perceptions and attitudes as well, as shown through the belief in high competency of employees with ASD; promotions; and supportive, positive coworker experiences. These results are promising for developing recommendations that can be implemented to increase employment opportunities for individuals with ASD on a large scale. More research, however, needs to be conducted to continue to identify best practices for supporting employees with ASD. As these recommendations are further researched and refined, employers will need training and support to translate these concepts into practice.

## Figures and Tables

**Table 1:** Accommodation.

Description	Survey Average
Regularly reviews the accessibility of its online application system to people with visual, hearing, finger dexterity, and cognitive impairments	61.0%
Analyzes our job descriptions to determine whether the responsibilities could be broken down into discrete tasks that could be performed by an individual with ASD	61.0%
Provides advance notice to job applicants that reasonable accommodations are provided during the job application process	68.0%
Evaluates pre-employment occupational screenings to ensure they are unbiased	71.0%
Has company-wide fund to provide accommodations for people with disabilities	61.0%
Has a designated office or person to address accommodation questions	69.0%
Has an established grievance procedure to address reasonable accommodation issues	70.0%

**Table 2:** Hiring.

Description	Survey Average
Hired someone with ASD in the last 5 years	58.0%
Actively recruits people with ASD	53.0%
Works with community organizations that promote hiring of people with ASD	57.0%
Includes people with ASD explicitly in its diversity and inclusion plan	58.0%
Has explicit organizational goals related to the recruitment or hiring of people with ASD	54.0%
Includes progress toward hiring goals for people with ASD in the performance appraisals of senior management	52.0%
Participates in internships that target people with ASD	53.0%
Has senior management that demonstrates a strong commitment to ASD hiring	56.0%
Utilizes tax incentives for hiring people with disabilities	54.0%
Requires subcontractors/suppliers to adhere to disability nondiscrimination requirements	56.0%
Does not automatically exclude job applicants with a history of unemployment	79.0%
Does not automatically exclude job applicants with a large gap in employment	76.0%
Has company initiative to hire people with HFASD	53.0%
Works with universities to hire people with HFASD	49.0%
Uses social media ads to recruit people with HFASD	45.0%

**Table 3:** Retention.

Description	Survey Average
Has a formal mentoring program to support employees with ASD	53.0%
Encourages flexible work arrangements for all employees with ASD (e.g., flextime, part-time, telecommuting)	62.0%
Offers special career planning and development tools for employees with ASD	55.0%
Has an ASD-focused employee network (e.g., employee resource group or affinity group)	52.0%
Invites employees to confidentially disclose whether they have a disability (e.g., staff surveys)	73.0%
Has explicit organizational goals related to retention or advancement of employees with ASD	54.0%
Includes progress toward retention of advancement goals for employees with ASD in the performance appraisals of senior management	54.0%
Allows an employee to exceed the maximum duration of medical leave as an accommodation	54.0%
Has defined career paths at our company for all employees	75.0%
Opportunities for advancement of employees with HFASD	61.0%

**Table 4:** Training.

Description	Survey Average
Offers ASD awareness and sensitivity training (internal)	60.0%
Offers ASD awareness and sensitivity training (external)	51.0%
Trains HR staff and supervisors on effective interviewing of people with ASD	62.0%
Trains HR staff and supervisors on inclusion practices of people with ASD in the workplace	62.0%
Requires training for supervisors on legal requirements of disability and non-discrimination and accommodation	68.0%
Includes ASD awareness and sensitivity as a topic in training for managers/ supervisors	58.0%
In contract with an agency that can help our business provide the support needed for working with employees with ASD now and in the future	52.0%

**Table 5:** Cluster average scores.

Cluster	Size	Accommodation	Hiring	Retention	Training
1	41	0.67	0.21	0.28	0.11
2	32	0.84	0.56	0.78	0.70
3	62	0.10	0.12	0.06	0.04
4	24	0.30	0.34	0.25	0.61
5	126	0.97	0.95	0.97	0.98

**Table 6:** Practices for improving employment of individuals with high functioning ASD.

Category	Practice	Supporting Citations
Recruitment and Hiring	Foster relationships with community organizations for recruitment of employees with ASD. Encourage relationships with universities and employment agencies to help bridge the gap between college and employment and allow for a smoother transition. Have a supportive interview process. It is not enough to simply note that accommodations will be provided during interview (e.g., Cluster 1); more is needed Have specific hiring and retention practices and policies related to ASD employees, along with training for managers, hiring personnel (specify who).	Culler et al. (2011); Fraser et al. (2010); Fraser et al. (2011); Gewurtz et al. (2018); Graffam et al. (2002); Peck & Kirkbride (2001); Wiggett-Barnard and Swarts (2012)
Training	Make hiring personnel, managers, and employees aware of the many positive professional traits that employees with ASD have; foster an inclusive workplace that reflects a belief in the high competency of employees. Provide training about the myths of ASD employment “challenges”.	Fraser et al. (2011); Lengnick-Hall et al. (2008); Lopez & Keenan (2014); Luecking (2011); Nesbitt (2000); Peck & Kirkbride (2001); Richards (2012)
Accessibility & Accommodations	Provide a menu of options, rather than just a few, that the employer believes are effective, for individuals to hire and retain employees with ASD. Offer for-credit internships; this creates more of an incentive to work there and can provide a transition opportunity for employees with ASD to adjust to the work environment. Provide the same professional growth opportunities for ASD as for neurotypical colleagues.	Project SEARCH [60]; Taube (2014) [61]
Retention & Advancement	Create and monitor a professional growth plan. Support employers (encourage ride-sharing, peer mentor/support, relationships of open communication and trust). Partner with or provide an on-site job training program.	Lindsay et al. (2019); Wiggett-Barnard and Swarts (2012)

## Abbreviations

The following abbreviations are used in this manuscript.

ASD	Autism Spectrum Disorder
HFASD	High Functioning Autism Spectrum Disorder
HR	Human Resources
